# Rehabilitation and release of confiscated songbirds into the wild: A pilot study

**DOI:** 10.3389/fvets.2023.1109568

**Published:** 2023-03-30

**Authors:** Cláudio Estêvão Farias Cruz, David Driemeier, Luciana Sonne, Saulo P. Pavarini, Welden Panziera, Gustavo R. Funkler, Nicole S. Böelter, Juan L. C. Homem, Camila E. S. Soares, Gabrielle Z. Tres, Vitor G. C. Silva, Miguel L. Correa, Francisco J. M. Caporal, Sandra T. Marques, João F. Soares, Paulo Guilherme Carniel Wagner, Walter Nisa-Castro-Neto, Inês Andretta

**Affiliations:** ^1^Centro de Estudos em Manejo de Aves Silvestres (CEMAS), Faculdade de Veterinária, Universidade Federal do Rio Grande do Sul, Porto Alegre, Brazil; ^2^Programa de Pós-Graduação em Ciências Veterinárias, Faculdade de Veterinária, Universidade Federal do Rio Grande do Sul, Porto Alegre, Brazil; ^3^Setor de Patologia Veterinária, Faculdade de Veterinária, Universidade Federal do Rio Grande do Sul, Porto Alegre, Brazil; ^4^Laboratório Porto Belo, Porto Alegre, Brazil; ^5^CMPC Celulose Riograndense Ltda., Guaíba, Brazil; ^6^Laboratório de Parasitologia Veterinária, Faculdade de Veterinária, Universidade Federal do Rio Grande do Sul, Porto Alegre, Brazil; ^7^Laboratório de Protozoologia e Rickettsioses Vetoriais, Faculdade de Veterinária, Universidade Federal do Rio Grande do Sul, Porto Alegre, Brazil; ^8^Centro de Triagem de Animais Silvestres (CETAS), Instituto Brasileiro do Meio Ambiente e dos Recursos Naturais Renováveis (IBAMA), Porto Alegre, Brazil; ^9^Organização para a Pesquisa e a Conservação de Esqualus no Brasil (PRÓ-SQUALUS), Tôrres, Brazil; ^10^Laboratório de Ensino Zootécnico, Faculdade de Agronomia, Universidade Federal do Rio Grande do Sul, Porto Alegre, Brazil

**Keywords:** *Atoxoplasma*, live decoys, financial costs, pathological findings, post-release monitoring, rehabilitation and release, seized passerines

## Abstract

Songbirds are currently the most prevalent animals in illegal trafficking in Brazil and other countries, so they are often confiscated, and this poses legal, ethical, and conservation challenges. Returning them to nature requires complex and expensive management, a topic that is sparingly addressed in the literature. Here, we described the processes and costs associated with an attempt to rehabilitate and release confiscated songbirds into the wild. A total of 1,721 songbirds of several species were quarantined, rehabilitated, and released, primarily on two farms located within their typical geographical distribution. Health assessments were performed on samples from 370 birds. Serology revealed no antibodies against Newcastle disease, and *Salmonella* spp. cultures were negative. Real-time polymerase chain reactions detected *M*. *gallisepticum* in samples from seven birds. *Atoxoplasma* spp. and *Acuaria* spp. infections, sepsis, and trauma were the top causes of bird death. About 6% of the released birds were recaptured, within an average period of 249 days after release, and at a mean distance of 2,397 meters from the release sites. The majority of these birds were found with free-living mates within or close to fragments of transitional ecoregions with native or cultivated grasslands, and native groves/forests, and shrublands. However, eucalyptus plantations with rich understory regeneration provided a suitable environment for the released forest species to settle, since they were recaptured during the defense of these sites. Over half of the recaptured birds presented behavioral profiles with both dominant and tame traits. Birds with dominant traits are more likely to settle in habitats and face the live decoys during fieldwork, whereas birds with tame characteristics tend to accept close contact with humans. Ultramarine grosbeak (*Cyanoloxia brissonii*), the least common species among those released, at the release sites showed an almost 2-fold recapture rate in the shortest mean distances from the release sites. This suggests less territory competition, perhaps a major factor of bird re-establishment here. The total per-bird cost was USD 57. Our findings suggested suitable survival and re-establishment of confiscated songbirds in the wild, when managed as we describe.

## Introduction

Spurred on by billion-dollar revenue, worldwide poaching and illegal trade remain top threats to wildlife survival, especially across the Americas, Asia, and Africa (1–3), where vulnerable rural communities are attracted to the potential source of income (4, 5). The demand for wildlife can be roughly grouped into four main categories i.e., collectibles, pets, traditional medicine/religion, and food (6). Birds, especially songbirds, are currently the most numerous species captured for live animal trafficking in Brazil (7), and elsewhere (6, 8, 9). While endangered songbird species are also trafficked in Brazil, most belong to common and widely distributed species with low conservation value (10, 11). Consequently, they are often confiscated and, despite their “least concern” rating on the ICMBIO and IUCN Red Lists (12, 13), this poses legal, ethical, and conservation challenges. In Brazil, these confiscated songbirds usually overwhelm the dedicated facilities and wildlife managers that receive them. Moreover, as there have been no recent or comprehensive population estimates, some least-concern species may in fact be in decline, due to uncontrolled trapping, the high demand for wild animal trade (14, 15), or other human threats (16, 17). Given that birds are crucial for balanced ecosystems and other ecological services (18–20), and songbirds constitute the majority of confiscations in Brazil and abroad, further strategies and management practices for their proper return to natural environments are needed. However, the decision to release confiscated wild birds should be made on a case-by-case basis and follow conservation guidelines (21, 22) that are based on genetic and health data as well as other studies (23–27). However, this information is scarce and conservation evidence on the subject is limited (28), especially concerning species that are not conservation flagships. Despite the high number of confiscations (6, 10), few reports about releases (29, 30) compete with the scores of vague disclosures in online media. Managing birds seized from illegal traffic is a complex and difficult issue (28, 31, 32) that remains sparingly addressed or undisclosed. In this pilot study, we described the processes and costs involved in an attempt to rehabilitate and release confiscated songbirds into the wild.

## Materials and methods

### General information

This project involved bureaucratic procedures (2015–2017), aviary construction (2017–2018), and bird management (2018–2022). The birds in this study were received from the Wild Animal Triage Center (*Centro de Triagem de Animais Silvestres*—*CETAS*) at the Brazilian environmental agency (*Instituto Brasileiro do Meio Ambiente e dos Recursos Naturais Renováveis*—IBAMA), located in Rio Grande do Sul (RS). While many other species composed the confiscated flocks, some of the most common and locally distributed species were selected for post-release monitoring, including the saffron finch (*Sicalis flaveola*), red-crested cardinal (*Paroaria coronata*), green-winged saltator (*Saltator similis*), ultramarine grosbeak (*Cyanoloxia brissonii*), and red-crested finch (*Coryphospingus cucullatus*) (songbirds group 1 – SBG1, *n* = 1,721) (Supplementary Table 1). The migratory double-collared seedeater (*Sporophila caerulescens*) (33), other species with lower representativeness, and those distributed at distant sites (songbirds group 2 – SBG2, *n* = 689) were not monitored after release (Supplementary Table 2). Although they were probably captured in RS (11), the origin of the birds was uncertain since they could have been illegally captured anywhere in their distribution ranges. At CETAS, large numbers of confiscated songbirds of the same and/or different species are usually kept in dozens of contiguous cages. Most are housed individually, but others remain in collective cages. Time in captivity can tame wild bird behavior (11). Before removing them from their cages to handle them, we observed their behaviors toward handlers to roughly classify traits into (1-tame) bird perching and feeding even when the cage was held by handlers, (2-semi-wild or undefined) bird perching and feeding when handlers remained about 5 m away from the cages, or when birds were housed in a collective cage, and (3-wild) bird fluttering, not perching or feeding when handlers remained about 5 m from the cage. Regarding their dominance behavior, such as vocalizations (repeated usual song, song switching, and long, loud whistles and calls) or fighting postures (ruffled chest and head feathers, fully raised crests, half-open beaks pointing upwards) (34) in response to playback of conspecific vocalizations or face-to-face conspecific challenges, birds were initially classified as (1-dominant) vocalizing and/or fighting postures, (2-semi-dominant or undefined) no vocalizing but also no signs of submission, and (3-submissive), mute birds with ruffled forehead feathers, lowered heads, retracted tufts, low chirping, and/or open lowered wings. Next, the birds were marked with split metal rings. Except for the red-crested cardinals and green-winged saltators that were ringed on both tarsi (to distinguish them from free-ranging songbirds already ringed and released) (27), all other species were ringed on one tarsus. After ringing, examining, sampling for health tests (15%), and medicating, the birds were allocated to individual transport containers and moved to the quarantine aviaries. The time between confiscation and transport to quarantine varied, depending on handler availability, but usually took 1 to 5 days.

### Health information

Any birds with clinical signs of dysfunction such as cachexia, diarrhea, accumulated droppings in vent feathers, eye or nasal discharge, conjunctivitis, dirty beaks, or even ruffled feathers were excluded from the quarantine flock and left at the CETAS for veterinary care. Pooled fecal samples were forwarded for parasitological tests after collecting them from plastic film left under cages for 20 min. During the health screening, all birds were examined for external parasites and proliferative skin lesions typical of avian pox. Rectrices and primary feathers were checked, broken feathers removed, long nails trimmed, and individual samples (feces, oropharyngeal swabs, and blood) collected, as described previously (34). Blood samples were only collected from birds weighing approximately 40 g. All the birds were dosed with Zooserine^®^ oral pills (tetracycline hydrochloride 0.18 mg/g, chloramphenicol 0.133 mg/g, and furazolidone 0.03 mg/g) and occasional tarsal hyperkeratosis was treated by manually applying Dolemil^®^ (potassium sulfide 3%) ointment. Terramycin^®^ powder with antigerm 77 (Zoetis – 2 g/L) and Panacur^®^ (MSD – Fenbendazole 200 mg/L) were added to their drinking water for three consecutive days each, during the second and third quarantine weeks, respectively. All doses served as preventive therapies to reduce protozoan and helminth transmission during captivity.

The hemagglutination-inhibition test for anti-NDV antibodies, *Salmonella* spp. cultivation, and real-time polymerase chain reactions to detect *M*. *gallisepticum* were performed as described previously (27). Budgetary constraints meant only about 15% of the confiscated birds underwent tests for pathogens. Aliquots from fecal sample pools were analyzed using Willis's flotation technique (35). Helminth eggs were detected, and all the oocysts on the slide were counted. Positive samples for oocysts were allowed to sporulate (36), and were then measured with the aid of an Ernst Leitz micrometric Wetzlar eyepiece. Remaining fecal content was analyzed using the Lutz method (37) for eggs and larvae. Except for the birds with an obvious cause of death (e.g., conspecific and predator attacks), all deceased birds underwent necropsy. During outbreaks, a 25%-bird sample underwent pathological examinations, and the probable cause of death was also attributed to the others. The carcasses were immediately submitted for necropsy, or frozen if they were discovered outside of laboratory hours. The necropsy remains and decomposing carcasses found in the rehabilitation aviaries were disposed of through a private medical waste removal service. Samples from several organs and tissues were collected, fixed, and processed in compliance with standard histopathological procedures. Hematoxylin and eosin, and other staining methods were used. Additional diagnostic immunohistochemical, and molecular assays were performed as required. Occasionally, sick birds were housed in separate cages and cared for as needed.

Total genomic DNA was extracted from necropsy samples (changed intestinal epithelium suggestive of *Atoxoplasma* spp. infection in 10 birds) using the PureLink Genomic DNA MiniKit (Invitrogen, Carlsbad, CA, USA). A nested PCR was used to test the samples. The primary reaction used outer EIMF (5′-ACCATGGTAATTCTATG-3′) and 990 (5′-TTGCCTYAAACTTCCTT-3′) and inner EIMR (5′-CTCAAAGTAAAAGTTCC-3′) and 989 (5′-AGTTTCTGACCTATCAG-3′) primers targeting a ≈ 455– bp fragment of the *Isospora* spp.18S rRNA gene, as previously described (38). Amplicons of the expected size of two positive samples, chosen at random, were purified with Purelink kits and Invitrogen^®^ reagents, and Sanger sequenced by ACTGene Inc. (Alvorada, RS, Brazil). Generated sequences were submitted to a BLAST search (39) to find regions of local similarity in the GenBank database. Partial sequences of the Coccidia 18S rRNA gene were aligned with corresponding 18S rRNA sequences of thirteen coccidian species, using Clustal/W v.1.8.1 (40), and an identity matrix was calculated using BioEdit software.

### Quarantine and rehabilitation

For quarantine, we reuse enclosures from a previous project (41, 42) located in the Ipanema district of Porto Alegre, RS. There are two interchangeable enclosures: (a) a 22 m^3^ fully roofed, aerial aviary with wire mesh floor and (b) a 72 m^3^ partially roofed, double-mesh outdoor planted aviary. Variable numbers of birds of mixed or same species and sexes, were primarily housed in (a), at least until we had verified their feather waterproofing status. The birds were housed according to their mean body weight (10–50 g), their nutritional habits, and/or stocking density ranges. Stocking density was managed by introducing birds until there was no more individual space available to defend and conflicts ceased. During quarantine, this usually resulted in densities between 4 and 7 birds/m^3^. Enclosures could also be subdivided with plastic mesh panels for adjusting bird densities. Persistent fighters were captured and relocated to enclosures with occupants of a higher body weight category, or temporarily isolated. Birds were checked daily to monitor conflicts, stocking densities, and feeding. They received a daily *ad libitum* diet of mainly seed mixtures (birdseed, millet, oat, sunflower, and rice), fruits (papaya, apple, orange, and banana), and vegetables (cucumber, green corn on the cob, lettuce, and cabbage). Bird baths were checked, cleaned, and exchanged several times a day. In cases of outbreaks or new arrivals, quarantine was reset to day 1. At around 30 quarantine days, if no disease was detected and there were only occasional traumatic deaths, the birds were caught, examined (physical condition, eyes, beaks, legs, nails, and feathers), placed in individual disposable cardboard boxes, and moved to the rehabilitation aviaries. Afterwards, the quarantine enclosures were cleaned (using steel brushes and a pressurized water jet) and left empty for 2–3 weeks before restocking with new arrivals. In the rehabilitation aviaries, the stocking density was also managed by monitoring conflict dynamics, but usually reached 0.5–1 bird/m^3^. Any aggressive birds were relocated, temporally isolated, or released. The recuperation period varied, depending on the response time to improved flock appearance (feathering) and behavior.

The rehabilitation aviaries were constructed on the edge of the riparian forest adjacent to the Dilúvio stream, at the Faculdade de Veterinária—UFRGS. Made from galvanized iron pipes, the structure was mounted on a 1 m concrete belt (0.8 m buried underground). A total of 1,000 m^3^ were distributed along two contiguous 5 m x 5 m x 20 m areas of fully planted (trees, shrubs, and vines) roofless enclosures. In the 200 m^3^ access barn, an additional 48 m^3^ were divided into two 2 m x 2 m x 6 m contiguous support enclosures. All enclosures were interchangeable. The main aviaries were bedded with a 25 cm layer of irregular 0.5 cm x 1.0 cm gravel and covered with a 15 mm x 15 mm galvanized welded wire (2.2 mm) mesh. Specimens of cambui (*Myrciaria tenella*), jaboticaba (*Myrciaria cauliflora*), Brazilian cherry-tree (*Eugenia involucrata*), Brazilian pitanga (*Eugenia uniflora*), cinnamon (*Cinnamomum verum*), littleleaf boxwood (*Buxus microphylla*), Buddhist pine (*Podocarpus macrophillus*), guaco (*Mikania glomerata*), and passionflower (*Passiflora alata*) were planted along the aviary landscape, and rocks and leafless tree branches were placed for perching. The diet during rehabilitation was similar to quarantine but offered every other day. Additional food items included live invertebrates (*Tenebrio molitor, Zophobas morio, Mocis latipes, Spodoptera frugiperda, Galleria mellonella*, grasshoppers, and spiders), branches/twigs with leaves and native fruits (*Allophylus edulis, Eugenia rostrifolia, Eugenia uniflora, Casearia sylvestris, Myrciaria tenella*, etc.) and grass inflorescences (*Avena sativa, Brachiaria decumbens, Cynodon dactylon, Lolium perenne, Panicum maximum*, and *Paspalum notatum*). Live invertebrates were confiscated, bought, and collected as described previously (43). Seed mixtures were served in roofed feeders and scattered around aviary grounds. Fruits and vegetables were mainly skewered onto branches. When birds started hiding within the vegetation upon our arrival at the aviaries, we planned the releases. After 2 weeks of no disease and only occasional traumatic deaths, the birds were lured with food into the contiguous barn enclosures, where they were manually captured (after dark), and placed into individual disposable cardboard boxes. They were kept there overnight, moved to release sites at dawn, and hard-released upon arrival, as shown in Supplementary Table 1. In addition to changing the 3-inch top layer of gravel bedding, the enclosures were cleaned with a pressurized water jet (for the lower structures), limed (calcium hydroxide – 0.5 kg/m^2^), and kept empty for 2–3 weeks before repopulation. Perching branches were replaced. The same cleaning procedures were performed in the outdoor quarantine enclosure.

### Release sites and fieldwork

The birds were released at sites along their usual range of distribution. Those from distant places (SBG2) were taken to them and fed until the time for release. For SBG1, release sites were planned up to 100 km from Porto Alegre, to accommodate long-term post-release monitoring. Additional criteria for selecting sites included long-term monitoring agreements with the farms, to guarantee access with an entrance key or a 24 h managerial service, and control human traffic. We did not perform any pre-release evaluations of the release sites. Farm 1 included a 1,200-hectare dual-purpose farm (crops and cattle) in the municipality of Eldorado do Sul. Farm 2 encompassed an 8,200-hectare eucalyptus plantation conjoining 2,400 hectares of native vegetation, mostly residual Atlantic forest and riparian shrublands at the edge of the Guaiba lake. Farm 2 lay adjacent to Farm 3, a 6,000-hectare dual-purpose farm. Both were located in the Barra do Ribeiro municipality. Occasional release sites included the surroundings of the rehabilitation facilities (CEMAS/UFRGS) in addition to a 30-hectare ranch in the rural vicinity of the Viamão municipality (Ranch1). At these release sites, green vegetables and fruits had been previously skewered onto nearby branches and seed mixtures scattered on the ground nearby. Combinations of native pastures, forest fragments, shrublands, cultivated crops, rural dirt roads with shrubby vegetation on both sides, eucalyptus plantations, and weir-adjacent or riparian vegetation characterized the release sites. Those habitats were appropriate for all species released and provided intersection of ecological corridors (i.e., dirt road crossed by a river) for bird dispersion. Having a nearby (up to 100 m from the site) body of water (e.g., dams, streams) was important for the bird's survival. Suitable release sites were not used while a released bird settled there.

Post-release monitoring was performed at least once a week, particularly during the breeding seasons of 2020, 2021, and 2022. Fieldwork was carried out between quarantine, rehabilitation and other activities. Post-release monitoring was mostly performed on the farms, where two project members covered 5 to 10 km a day. To attract target species and recapture released birds, we used live decoys, mist nets, cages fitted with netted trapdoors, and loop snares, as previously described (34). Recapture was necessary for identifying ring numbers and assessing recorded data (entry, release, and distance moved from release site, Supplementary Table 1). Recaptured birds were released immediately after identification. The live decoy cages were placed on the luggage rack of a vehicle that traveled slowly along each farm's road networks, crops and cattle grasslands. Each day, once there was vehicular access, we drove close to the coordinates of the last release or recapture and continued along available routes. In addition, we also followed tips from local people about the sightings or location of ringed birds. After hearing or sighting a target species and using binoculars to verify ringed subjects, the capture arena was installed (34). If a target bird approached the car very quickly, we simply installed netted trapdoors and snares. After installing the capture arena, it was constantly monitored to prevent opportunistic predator attacks and minimize additional stress for the captured birds and decoys. For each arena where we recaptured a released bird (positive), other attracted birds (negatives) were also recorded. Ringed birds that came within sight but did not approach were disregarded, as well as the unidentified negatives. Since large eucalyptus plots can present an undistinguishable landscape, we used the tracking tool from Avenza maps. Approximate release and recapture coordinates were obtained using the Google Earth geobrowser and the distances between them were calculated with the measuring tool.

### Financial information

Most of the project acquisitions and payments were performed by the team from the Federal University of Rio Grande do Sul's support foundation (*Fundação de Apoio da Universidade Federal do Rio Grande do Sul*—FAURGS). Expenses were recorded under different categories, as presented in Supplementary Table 3. All the expenses were incurred in Brazilian reais during the term of the project and converted to the commercial dollar value on the payment date. To calculate the final cost per bird, all expenses were included, except publications costs. Building costs over 10 years (the minimum aviary lifespan) were similarly included into the 4-year recuperation period (2019–2022). Bird management was carried out by CEMAS staff (*n* = 5) who were not paid through the project. We estimated a minimum wage for quarantine/rehabilitation of USD 5.00 per hour, and calculated work time as 3 h/day during quarantine (30 days) and 3 h every other day x average rehabilitation period/2 (30 days) x number (*n* = 11) of bird flocks—(line 46, Supplementary Table 3). We attributed USD 90.00 per day for post-release monitoring (line 47, Supplementary Table 3), the average of daily values budgeted by 2 local biologists. Most financial support was provided by CMPC Celulose Riograndense Ltda. (column C, Supplementary Table 3), but the project also received grants from the coordinator and IBAMA (column D, Supplementary Table 3).

### Data analysis

For fieldwork data, descriptive variables for each SBG1 were recorded in a spreadsheet (Supplementary Table 1). Responses were expressed using descriptive statistics. Pearson's chi-square test was used to assess the association between categorical variables. Variance analyses were performed using the General Linear Model procedure and Tukey's test was used to explore differences among the means. The 95% confidence intervals (CI) were calculated using a one-sample proportion test. The analyses were conducted with Minitab v. 20 software (State College, Pennsylvania, USA. http://www.minitab.com) and interpreted at a significance level of 0.05.

## Results

### General songbird management

**Figure 1:**
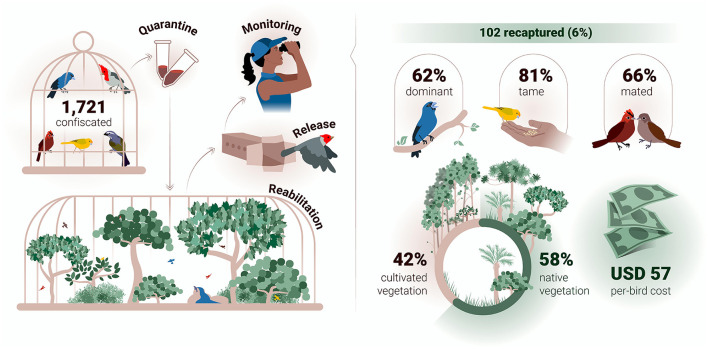
Graphical Abstract.

In the period October/2018—October/2022, a total of 1,721 songbirds (SBG1) comprising 642 saffron finches, 335 red-crested cardinals, 310 green-winged saltators, 307 ultramarine grosbeaks, and 127 red-crested finches were quarantined, rehabilitated, and released (Supplementary Table 1, Figure 1). The mean quarantine-rehabilitation period was 89 days (median: 82 days; first and third quartiles: 75 and 100 days). Over 215 monitoring days distributed over the 2019–2022 period, and performed mostly during the reproductive seasons, a total of 102 (6%) out of 1,721 songbirds (SBG1) were recaptured an average of 249 days after their release (median: 186 days; first and third quartiles: 53 and 377 days), at a mean distance of 2,397 m from the release sites (median: 1,370 m; first and third quartiles: 698 m and 3,360 m).

**Figure 2:**
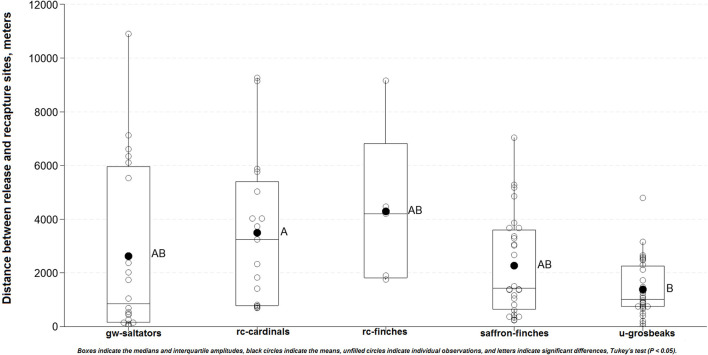
Distance traveled between release and recapture sites by the songbird species.

The period from release to recapture was similar among the bird species (*P* = 0.722). However, the dispersal of recaptured birds differed significantly between species (F = 3.207; *p* = 0.016) (Figure 2). The red-crested cardinals were recaptured at further mean distances (3,487m) than ultramarine grosbeaks (1,386 m; *P* = 0.009). In addition, the red-crested cardinals had shorter quarantine-rehabilitation periods (84.3 days) than ultramarine grosbeaks (96.1 days; *P* < 0.001). Other species showed statistically intermediate means in both assessments. Low correlations were found between the quarantine-rehabilitation and release-recapture periods (*r* = −0.101), and between the latter (*r* = 0.088) and the former (*r* = −0.307) periods and the release-to-recapture distance. The Pearson's chi-square test showed no association (*P* > 0.05) between the chance of recapture and the duration of quarantine-rehabilitation period. Most recaptured birds were both dominant (62%), and tame (81%), and were attracted/captured during territory defense (78%). At recapture, 68 (66%) of them were observed with free-living (*n* = 54) and rehabilitated (*n* = 14) mates. Birds' dominance behavior was closely associated (*P* < 0.001) with their tame behavior, while both behaviors were associated with sex (*P* < 0.001), and species (*P* < 0.001). The chance of recapture was associated with the bird species (*P* = 0.005), sex (*P* < 0.001), dominance behavior (*P* < 0.001), and tame behavior (*P* < 0.001).

Most recapture environments were transitional ecoregions including native and cultivated grasslands (46%), eucalyptus plantation understory regeneration (38%), native shrublands (35%), and native grove/forest fragments (34%) (Supplementary Table 1). The distance release-to-recapture (*P* = 0.005), season (*P* = 0.013), and environment type (*P* < 0.001) were associated with the recaptured species. The ultramarine grosbeak showed a higher recapture rate (10.7%), and the shortest mean distances (1.400 m) from the release sites. Additional recapture rates per species included the green-winged saltator (6.1%), red-crested cardinal (5.3%), saffron finch (4.5%), and red-crested finch (3.9%). A total of 1,094 negative birds from 77 species were recorded. The most prevalent negative birds were saffron finches (*n* = 207), rufous-collared sparrows (*Zonotrichia capensis*) (*n* = 159), green-winged saltators (*n* = 130), and red-crested cardinals (*n* = 114). Other conspecific negatives were identified as 93 red-crested finches, and 28 ultramarine grosbeaks (Supplementary Table 1). The species of the negative birds were associated with the recaptured species (*P* < 0.001). In total, 63 (out of 965) and 39 (out of 756) recaptured birds were released in spring/summer and fall/winter, respectively. Released birds traveled mean distances of 2,397 m in spring/summer, and 2,472 m in fall/winter. Twenty-four ringed birds were sighted but not recaptured. Nine birds escaped during management, 5 of which were recaptured.

### Health tests and pathology

Serological testing revealed no antibodies against Newcastle disease. *Salmonella* spp. cultures were negatives. Of the collected samples, 2.1% tested positive to an *M. gallisepticum*–specific PCR (0.75–3.83% CI) (data regarding the health tests are presented in Supplementary Table 4). Real-time polymerase chain reactions detected *M*. *gallisepticum* in the samples of four red-crested cardinals and three green-winged saltators. Typical sporulated *Isospora* spp. oocysts (*n* = 11) with 2 sporocysts each measured 16–21 μm x 17–22 μm. Oocysts presented smooth double walls. Deaths occurred mostly during the first 3 weeks of quarantine. The findings on bird losses are summarized in Supplementary Table 5. Supplementary Table 6 shows the Atoxoplasma matrix. Sequence 1 showed 100% coverage and 97.88% similarity with *Isospora lunaris* (Access: MT237177.1). Sequence 2 showed 100% coverage and 97.72% similarity with *Isospora* spp. (Access: MH698576.1) and *Atoxoplasma* spp. (Access: AY331571.1).

### Financial costs

Except for publishing costs, the total cost for the research period was USD 137,312.66 (Supplementary Table 3). This sum was divided by the total of 2,410 released songbirds (SBG1 and SBG2) resulting in a per-bird cost of USD 56.98. Without post-release monitoring, the estimated per-bird rehabilitation and release cost decreased to USD 37.

## Discussion

While the treatment and temporary care of injured, diseased, or displaced indigenous animals, and their subsequent release into the wild, have been described (44, 45), detailed descriptions of the protocols behind rehabilitating and returning confiscated songbirds to the wild are seldom published or remain undisclosed. There is, therefore, a huge discrepancy between the reports of bird confiscations (6, 10) and those referring to management practices for properly returning (21) the birds to the wild (29, 30, 46). As such, one cannot rule out the possibility that most birds are being managed and released improperly. Translocation and post-release monitoring of captive-bred birds are sometimes employed as the only options for recovering endangered species (47–51). However, the species included in this study are classified as “least concern” birds on the ICMBIO and IUCN Red Lists (12, 13). Most conservation efforts do not address species unthreatened with extinction (13), yet these are the animals that constitute the bulk of confiscations in Brazil (10) and worldwide (8, 52), the impacts of the decreasing abundance of these common species are a growing global conservation concern (53–55). The loss of their ecosystem services could lead to greater repercussions than those of rare species. Moreover, there are relevant animal welfare challenges to consider. The appropriate management of confiscated songbirds is an urgent priority for wildlife professionals and policymakers from countries where high rates of illegal wild songbird trafficking are routine.

### Songbird management

To give confiscated birds a suitable second chance, rehabilitation should focus on providing the basics (food, environment, and health management) until they are healthy enough (body condition, plumage integrity, and behavior) to face life in the wild. The recuperation period should be as short as possible since delayed release may increase deaths (31) and costs (44). Another reason for ending captivity promptly is the difficulty in providing them with the full diet they have access in the wild. Although we included several types of foods, the readily-available diet in the wild is quite diverse, and they can consume large amounts of items like live invertebrates (56). This predatory habit increase captive-bird breeding (56, 57) and probably expedites rehabilitation also. Suitable access to sunlight and rain was available in the rehabilitation enclosures, so that feather quality clearly improved during the recuperation period. Healthy feathers assist with body thermoregulation and flight (58, 59). The birds immediately took repeated and frenzied baths upon having access to water, which reflected the long-term neglect of feather care. When their feathers do not become waterlogged after bathing, it is a sign that birds are ready to be moved to open roof aviaries. Flight distance and sunlight are important aspects to consider when planning the aviary dimensions and location. Our aviaries allowed flight distances about 100x the average body length of the housed birds, and the flight patterns upon release suggested suitable muscular conditioning. Growing healthy, lush, and adequate aviary vegetation provides comfort, shelter, and food for the birds (56), and is the main reason for letting as much sunlight as possible into the outdoor enclosures. The aviary stocking rates depended on number of attacks, persecutions, and ceased conflicts. Overcrowding leaves no available individual space to fight for. This is also a routine practice to promote peaceful coexistence among clownfish in captive-breeding (60). Controlling stock density was key, as the fourth cause of death was conspecific attacks (Supplementary Table 5). Considering the great variability in the number of confiscated songbirds over time (11), and conflict dynamics, it is useful to install multiple enclosures (preferably with adjustable dimensions) interconnected by a service corridor/interchangeable area (56). Conflict dynamics may be circumvented as reallocation to a contiguous aviary may establish a new flock hierarchy. In addition, if used for quarantine, these enclosures should also be installed with double-panel meshes (anti-predator) and aerial mesh floors. Despite the appearance of opossums (*Didelphis albiventris*), roadside hawks (*Rupornis magnirostris*), and other predators in the rehabilitation enclosures, there were still more advantages than disadvantages to the rehabilitation program. However, the aviary should be made rodent-proof (with concrete foundations and reinforced small mesh panels) since rats (*Rattus rattus*) are top songbird predators (56). Comparable findings (61) highlight that trauma prevention is crucial, especially during transport and reception, but also during the critical initial (62) period of quarantine. Disposable cardboard boxes provide a hygienic, dark (to keep birds quiet), and safe environment for temporarily placing (63) and transporting (56) songbirds.

### Health, disease, and pathology

Wild birds will do their best to mask signs of disease as a basic survival behavior (64). The presence of pathogens may be influenced by factors such as diet, environment, general health, and co-infection (65). Alone or together, and to greater or lesser extents, all these factors probably affect confiscated birds. As such, by selecting apparently healthy songbirds, we could hypothetically minimize disease prevalence during the recuperation period. This brings us to the importance of a comprehensive disease screening protocol, which is typically informed by lifelong practice and research on the health management of songbird flocks. Ideally, the decision making should also include the current health status of the free-living bird communities in the release sites. However, does one need to worry about pathogens in confiscated birds if their incidence in the free-living birds in the release sites is unknown? The answer is unclear. As discussed previously (27), despite the range of diseases affecting wild birds (66), we opted to investigate only those addressed by the National Plan of Avian Health because they may be important infectious conditions in both wild and commercial birds. Negative anti-NDV antibodies and *Salmonella* spp. culture (Supplementary Table 4) results corroborated a tendency we observed earlier (27). Comparable results have been reported in NDV serological surveys involving captive (67) and free-living wild birds (68). While previous studies have estimated the prevalence of *Salmonella* spp. in samples of wild birds – including passerines confiscated from illegal traffickers – at 1–7% (66, 69), a similar project (61) reported the same salmonellosis prevalence as we did. The PCR-based estimate of a 2.1% *M*. *gallisepticum* (MG) prevalence is lower than in our first report (27) but remained similar to findings reported for other avian hosts (70). We detected MG in samples from seven birds from one confiscated flock (Supplementary Table 4), and two MG-positive cardinals became ill and died from a condition indistinguishable from MG. The remaining flock was returned to CETAS for veterinary care and/or reallocation to authorized commercial captive breeding centers.

**Figure 3:**
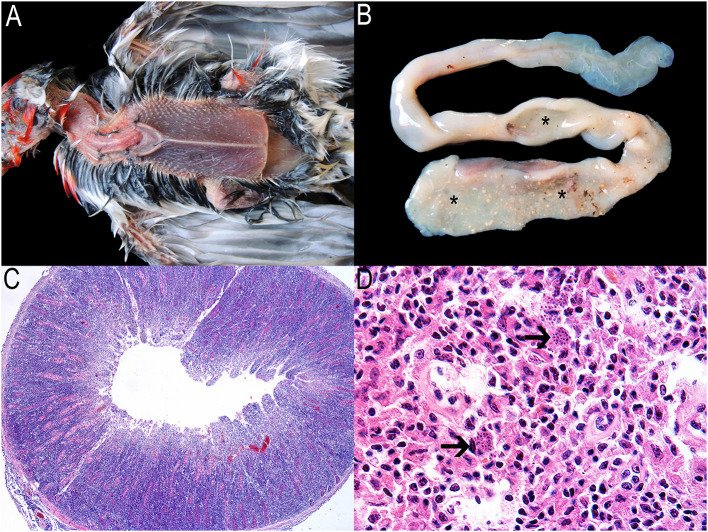
Atoxoplasmosis associated with lymphoproliferative diseases in songbirds. Macroscopic and microscopic findings. **(A)** Red-crested cardinal in poor physical health evidenced by marked atrophy of the pectoral muscles. **(B)** Evident thickening of the initial portion of the small intestine (asterisks). **(C)** Small intestine. Intense lymphocytic proliferation obliterating the entire intestinal mucosa and sometimes extending to the serosa. Hematoxylin and eosin 40X. **(D)** Spleen. Numerous small merozoites within the cytoplasm of macrophages (arrows). Hematoxylin and eosin 1,000X.

In total, the findings of 111 (67%) of the pathological examinations performed on 180 bird carcasses suggested disease. Atoxoplasmosis (Figure 3) and acuariasis were among the most prevalent postmortem findings, mainly in red-crested cardinals and seedeaters (*Sporophila* spp.), respectively (Supplementary Table 5). These infections and the prevalence of *Isospora* spp. in fecal samples (Supplementary Table 4) highlight the high risk of gastrointestinal parasites disseminating *via* the fecal-oral route in captive wild bird management projects (71). As such, housing the birds in enclosures with aerial mesh floors is recommended to minimize transmission (42), especially during quarantine. Systemic isosporosis, also known as atoxoplasmosis, has been referred to as a significant cause of mortality in captive passerines (72, 73). The debilitating and fatal disease occurs in association with stress, concurrent infections, or immunosuppression, conditions often affecting illegal trafficked birds (61, 74). However, *Atoxoplasma* spp. infection in free-ranging birds may not result in a significant mortality rate. Affected birds show prominent keel bones and severe pectoral muscle atrophy associated with thickening of the small intestine (75) due to lymphocytic proliferation (Figure 3). At least two confirmed outbreaks of the infection primarily affected two flocks of *Paroaria coronata*, although it spread to some *Saltator* spp. birds as well, especially *S*. *similis*. Both are species in the Thraupidae family, which adds information to the families most at risk, besides Fringillidae and Sturnidae (72).

The ulceration and inflammation of the gizzard mucosae due to *Acuaria* spp. infection may lead to subsequent digestive obstruction and death due to secondary infections or starvation (72). Comparable mortalities due to *Acuaria* spp. infection have been described for finches (76) and young swans (77). We reported a fatal infection in a pekin robin (*Leiothrix lutea*) (62) and afterwards we found *Acuaria* spp. in woodlice samples (43). *Acuaria spiralis* infections in wild birds have been attributed to the ingestion of parasitized woodlice (72). For unknown reasons, except for two passerines, this infection was restricted to small (10 g bw) seedeaters (*Sporophila* spp.). Perhaps dietary habits, specific nutritional needs, unique sensitivity, or other factors may explain it. The wide distribution of woodlice favors occasional contact and disease, especially in birds managed in outdoor aviaries. Moreover, the desirable calcium content (78) may encourage uncontrolled consumption by captive birds, usually those deprived of a balanced diet. Several other parasites were observed (Supplementary Table 5). Microfilariae were frequently detected, but they were not directly associated with the main cause of death. Possible associations with pulmonary hemorrhages may have included sarcocystosis (79) and vasculitis due to microfilariae (72), or other factors. Given possible self-medicating behavior of wild animals (80), concerns regarding these infections apply more to birds held in captivity.

Several wild animals suffer from captivity stress, which may be associated with diseases, deaths, and other conditions (81–83). Diverse opportunistic bacteria can cause sepsis (66), a common infection complication leading to death in confiscated birds (61). Sepsis was also observed in our subjects, as well as aspergillosis and candidiasis which are expected diseases among songbirds undergoing the usual stressors in illegal trafficking. Wild birds may also die from acute stress-induced cardiogenic shock due to decreased circulating plasma, physical restraints, or hypovolemic shock resulting from insufficient blood volume caused by acute hemorrhage or excessive fluid loss (34, 84). Given the fighting dynamic we already mentioned, we could plausibly attribute death to cardiogenic shock to explain some of the inconclusive losses with no inflammatory or infectious findings (Supplementary Table 5).

Gut dysbiosis is characterized by the loss of beneficial microbiota, altered epithelial permeability, and increased susceptibility to infection (85). The condition also affects wild birds (86), and could be a plausible cause of death in the group of inconclusive necropsies, especially those in which the main changes were restricted to the intestines. Songbird carcasses have often been associated with considerable autolytic changes (73, 87) and high rates of inconclusive post-mortem findings (61). Rapid autolysis of these carcasses may result from high body temperature (40°C) and body heat insulation (88).

### Fieldwork

To assess the rehabilitation and release program, it was crucial to evaluate survival and re-establishment. The high costs of better quality camera lenses, short radio transmitter battery lifespans, other expensive devices (89) and basic photography training limited our budget for GPS units and cameras, to use as alternative recovery methods. As a priority we released birds on properties where the owners, supervisors, and workers, had long-standing, trusting relationships. Naturally, the birds may have eventually traveled beyond farm boundaries. However, we tried to ensure their safety for the first hours after release, when they are most likely to be vulnerable to illegal recapture. Most hard-release sites transitioned from grasslands to riparian vegetation and could provide food, water, shelter, and ecological corridors for the birds (90). Feeding areas where large wild flocks band together, particularly during winter months, also served as release sites. Alternative criteria for selecting release sites instead of those based on population genetics (largely unknown for most of these species) include (a) proximity to the confiscation sites, for the immediate release of freshly-caught wild birds, and (b) different regional vocal “dialects” as a proxy for the original parental population of a songbird (91). Nevertheless, although interesting, these alternative criteria can be difficult or impractical for managing confiscated songbirds. This is because, except for the top dominant ones, most songbirds will not vocalize right away. For them to do so, they would have to be kept individually in cages, under reasonable level of care, for several days to weeks.

While most paths in the release sites were explored several times, higher numbers of recaptures were recorded along the access paths, especially of Farm 2 (data not shown). As these were the most frequently traveled paths, more fieldwork might have resulted in higher recapture rates, as was previously suggested (90). Placing the live decoy cages on the luggage rack allowed us to cover several territories more quickly. As soon as the decoys detected the conspecifics, they started to vocalize, so we stopped and checked for rings on the attracted bird(s). SBG2 species (Supplementary Table 2) were excluded from the post-release monitoring due to a lack of resources for long-term fieldwork at long distances. Besides this, it would have meant taking care of an even larger live decoy flock. We have not found any reports describing the sampling and managing of confiscated songbirds as we have done here. Similarly, we found no reports about using live decoys as a post-release monitoring tool, probably due to current legislative, regulatory, ethical, and logistic challenges (34). Decoys with a marked tame and dominant profile proved effective in the field for many reasons, including their connection with handlers, and level of care. Despite some challenges (34), we are convinced that these few passerines may have served the welfare of hundreds that were given a second chance, and possibly thousands of confiscated songbirds in the future.

While our 6% recapture rate is comparable to the overall average recovery rate (RR) of 7% of ringed and recovered free-living birds, in North America and Europe, where banding has existed for over 100 years (92–94), these records reported significant species diversity, and described various methods. In the absence of a closer reference with which to compare our data, it could, at least hypothetically, suggest comparable survival rates. Higher RR rates, median times elapsed from release dates, and median distances traveled between ringing and recovering were recorded for non-rehabilitated non-passerines than rehabilitated ones (95). Unlike that study, we excluded sick and injured birds. We also carried out most of the recaptures within the first year (95). Continued, long-term monitoring may change this, as we were able to perform some recaptures after the first year of release, particularly in places with a heavier human presence (L.12 and L.216, Supplementary Table 1). Again, these findings suggest that more fieldwork could result in stronger data. A study more similar to ours (46) investigated a smaller sample of 4 pairs of wild-caught Sumatran laughingthrushes (*Garrulax bicolor*). Residents had surrendered the birds to the Indonesian Species Conservation Program Center, where they were quarantined and rehabilitated over 90 days, and soft-released. Only one bird was tracked for the minimum 21-day battery lifespan, because the others transmitter signals were lost. Previous results suggest there may be some misconceptions about the benefits of delayed release, also called soft-release, which had a negative effect on the long-term survival of 59 threatened New Zealand hihi (*Notiomystis cincta*). They were wild-caught, screened for diseases (96), and fitted with radio transmitters before releasing (31). While some threatened cirl buntings (*Emberiza cirlus*) were removed from their nest, hand-reared and then delayed-released (97) in spring/summer, and were more likely to survive than those released afterwards (98), we did not observe differences between the recapture rates of birds released in different seasons.

Most of our recaptures occurred during the breeding seasons (78%), when most songbirds are prone to fight with the decoys while defending territory. Most of the recaptured birds were both dominant (62%), and tame (81%), and this probably reflects a biased result associated with our fieldwork methods. Considering that most of the birds we released were neither dominant (*n* = 1,236) nor tame (*n* = 1,196) (Supplementary Table 1), they probably would not be willing to face the decoys or allow humans to approach them. Birds with dominant traits drive away conspecifics while those with tame characteristics allow humans to approach them. Both are recognized songbird characteristics among bird keepers (7, 99). Presumably for the same reasons, we could not recapture any of the 24 ringed birds that came into sight but did not approach. For others, we did not have appropriate conspecific decoys. These aspects may explain why almost half of the recaptured birds belong to forest species that usually defend their territory all year round, even more so during the breeding season. Both the saffron finch and the red-crested cardinal are open area birds that usually form large winter flocks (100) to comb the countryside in search of feeding areas. This behavior may partially explain why, despite being among the most numerous groups, their recapture numbers were lower than those of forest species. Additionally, the largest monitored areas were covered with eucalyptus plantations. Most of the confiscated passerines that we could sex were male (*n* = 845), which are also usually the best and most valued singers among bird keepers.

Rather than considering individual characteristics, we based our decisions to release on general flock appearance and behavior. Most of the recaptured birds also looked healthy, with perfect feather cover, good physical status, and wild behavior. Even those that had been tame seemed wild at the first moments of reencounter. However, after several minutes, some of them seemed to get used to us, or ignored us to scare the decoys away. Contrary to what we used to think, even tame birds can survive and apparently adapt to free living (Supplementary Table 1), probably also due to the phenomenon referred to as social facilitation or observational learning (101). While our study focused on bird flocks, some individual results have challenged our perceptions. Firstly, even after 19 months in freedom, a pair of ultramarine grosbeaks (L.262 and L.264, Supplementary Table 1) were still tame enough to perch on car doors. Also, settling in a storage area for road maintenance materials suggests that they enjoy the human company there. Secondly, a male saffron finch made an impressive recovery (L.1577, Supplementary Table 1) and, in just 30 days post release, had mated and produced 2 fledglings. This family used an unoccupied rufous hornero nest in an 8m high eucalyptus tree.

A reintroduction of about 400 wild-caught North Island robins (*Petroica longipes*), which were immediately released upon arrival at the site, showed that the landscape connectivity, predator control, and forest type were strong predictors for post-release establishment (97). The specific factors underlying the re-establishment or dispersal of our released birds remain unclear. However, our findings suggest that available territory was the most important factor for songbird re-establishment. Ultramarine grosbeak (*C*. *brissonii*), the least common species (among those released) at the release sites (Supplementary Table 1) accounted for nearly double the overall recapture rate (10.7%), and the shortest mean distances (1.400 m) from the release sites (Figure 2), suggesting less territory competition. In addition to proper settlement, the majority of released ultramarine grosbeaks mating with free-ranging females also suggests a reduced male population. This, in turn, may suggest the illegal capture of males since they are more valued in trafficking (7) and are also more easily captured, since territory defense is usually a male task (11, 34). This situation, which may progressively affect a wider range of species worldwide, has been reported and linked to low reproductive success in the free-living populations of the endangered yellow cardinal (*Gubernatrix cristata*) (102). Enhanced dominant behavior may be another factor to predict re-establishment for some from our birds. However, as also discussed here, this may be biased data. In addition, we speculate that low competitiveness in the eucalyptus plantations (103) could also facilitate the re-establishment of released birds.

Our releases were performed along landscapes that provided birds with a functional connection between habitat patches to facilitate dispersal (90), an important requirement for common and widely distributed species. Most recaptured birds were found in environments which included native vegetation remnants within grasslands, shrublands, and groves or forest patches. A balanced conservation effect of preserved fragments and environments is essential (19, 20). Losses of natural and semi-natural habitats, mostly to agriculture, are a significant concern for biodiversity. While natural forests are progressively decreasing, plantation forests are increasing (104, 105). The critical effects of agricultural expansion on wildlife conservation point to a need for urgent, comprehensive agricultural planning (106), particularly in countries where considerable wildlife resources still exist. With releases in eucalyptus plantations, we expected to provide the released birds with a transient, sheltered, and less competitive environment than native forest fragments (103). However, a considerable number (38%) of our recoveries settled within eucalyptus plantations, especially in areas with rich understory regeneration. We observed the released birds and other free-living ones feeding on pioneer trees interspersed among the natural regeneration of bugre herb or wild-coffee (*Casearia sylvestris*), capororoca (*Myrsine umbellata*), and capulin (*Trema micrantha*). We also observed them feeding on wooly palm (*Butia* spp.), queen palm (*Syagrus romanzoffiana*), fig tree (*Ficus* spp.), and signal grass (*Brachiaria decumbens*), among other plants composing those landscapes. Natural regeneration establishes itself ~18 months after eucalyptus planting, when growing vegetation is no longer controlled. These findings are consistent with previous reports showing that afforestation of agricultural lands may assist conservation by providing complementary forest habitats. Considering these wildlife opportunities exist alongside deforestation, the pressure for agricultural development may render plantation forestry a “lesser evil” (104), especially if forest managers protect indigenous vegetation remnants (104). Our results corroborate that remnant patches of native vegetation (104), strips of riparian vegetation, dams, open and clearing areas (Supplementary Table 1) can increase the number of native species that occur on plantations (103). The harvesting of eucalyptus, usually after 7–10 years, could become a critical question here, since settled birds are obligated to look for new territory. However, the post-release period spent in the wild until harvesting may teach valuable skills to these birds, making them stronger than they were at release and able to venture further. They may learn to move on to the next eucalyptus plot as we observed with one ultramarine grosbeak pair (L.524, Supplementary Table 1). Considering the succession of stressful events to which these confiscated birds were subjected, it is reasonable to think that moving due to eucalyptus harvesting may not be harder to deal with than everything else they have been through.

### Financial costs

Financial records on managing wild birds are scarce, even more so for rehabilitating confiscated wild birds. However, unlike our case, the accounts for rehabilitating and releasing oiled wildlife on a per-bird basis started with a minimum of USD 1,600.00 (107), regardless of the construction costs, and volunteer work. These were excessively higher than our costs, greatly due to the medical expenses for injured birds, a category we did not include. A per-bird cost of USD 2,800.00 was estimated to reintroduce yellow-shouldered Amazon parrots (*Amazona barbadensis*) on Margarita Island, Venezuela (47). Previously (41), we discussed that, though construction may be initially expensive, these costs are absorbed over the aviary lifespan and highlights the importance of investing in durable materials, as we did. Our rehabilitation enclosures will last longer than the estimated 10-year lifespan. In that report, it was also made clear that live food, although highly appreciated by the birds, and possibly having high rehabilitation value, is hardly affordable in the amounts they usually consume. We mentioned an annual cost of USD 294.00 for a 20 g bw pekin robin (*Leiothrix lutea*), of which USD 259.00 was spent on live insects (41). Those were just a few songbirds kept at a high level of care that is unsustainable for large numbers of confiscated passerines. Also, unlike the study of oiled birds (107), in which one-half of the total costs paid for staff, we estimated our labor costs based on the minimum wage. We worked from the construction to the post-release monitoring phase. Except for daily personal expenses (L.30, Supplementary Table 3) on long trips, we received no payment through the project. Although studies tend to not address expenses, authors who are aware of the cost-effectiveness involved in rehabilitating injured birds have discussed the applicability of euthanizing difficult cases to save resources for the birds that would probably survive (45).

### Concluding remarks

While thousands of wild songbirds are confiscated annually in Brazil (7, 11, 99) and abroad (3, 9) these registered cases are probably only the tip of the iceberg. Managing songbirds confiscated from the illegal trade is controversial, complex, and often frustrating due to the many deaths, inconsistencies and questions associated with conservation effects, not to mention the related risks (24, 27, 32, 71, 74). However, it seems reasonable to think that losing thousands of them every year may impact nature more severely than this method of confiscating, recuperating, and returning birds to the wild, in compliance with guidelines (21, 22). Nevertheless, the long-term effectiveness of this conservation tool remains to be seen. We understand that much remains to be clarified about the genetic and health impacts involved. To date, studies using samples from three of the most commonly trafficked species in Brazil (24, 27) concluded that a better understanding of population connectivity among and within ecoregions is necessary to evaluate the feasibility of releasing confiscated birds in the wild. Considering the number of confiscated songbirds and the wide distribution of some trafficked species, significant effort and resources are needed to properly decipher these gaps. In the face of limited data, questions regarding the most feasible destination for these birds remain unresolved. While we may have taken several steps forward on management practices, establishing a definitive health screening protocol requires further management and research. Our pathological studies indicated that basic bird management practices, strict parasite control and appropriate enclosure design may prevent most bird losses. Above all, quarantine installations should include aerial mesh floors, double-mesh panels, and interchangeable aviary units to minimize fecal-oral transmissions, predator attacks, and aggression issues, respectively.

Although some aspects of captive management and the reestablishment of ecological services remain unclear, animal welfare and the ethical value of providing the birds with a suitable second chance were undoubtedly addressed. Songbirds suffer the same fate in many parts of the globe, some even more intensely where caging birds is a cultural habit (6, 10). The present report describes management practices for the appropriate rehabilitation and release of songbirds confiscated from illegal traffic. Further, we showed that, if managed as we describe, they may survive and re-establish themselves in the wild. Moreover, similar recovery rates indicate that their quality of survival may compare to that of free-ranging birds in the wild (92–94). Apart from being applicable in similar initiatives worldwide, these methods may also assist with confiscated songbird species of vulnerable conservation status, such as the great-billed seed-finch (*Sporophila maximiliani*) (108), also present in confiscations (11). While natural forests are obviously more suitable as habitat for a wider range of native forest species than plantation forests (20, 103), we observed that eucalyptus plantations provided suitable habitats for rehabilitated and released forest species, particularly in plots with more lush understory regeneration. In the past, eucalyptus plantations may have been considered “green deserts” (105), but this label may no longer be fitting in our current, changing world.

## Data availability statement

The datasets presented in this study can be found in online repositories. The names of the repository/repositories and accession number(s) can be found in the article/Supplementary material.

## Ethics statement

The study (no. 23644) was approved by the UFRGS Animal Ethics Committee and licensed by the *Instituto Chico Mendes de Conservação da Biodiversidade* (ICMBio), under license number 37567.

## Author contributions

CC, DD, and PW: conceptualization. CC and PW: funding acquisition and supervision. CC, NB, JH, CS, GT, VS, and MC: bird management. CC, NB, JH, CS, GT, VS, MC, and FC: fieldwork. LS, SP, WP, GF, SM, and JS: laboratory work. WN-C-N and IA: data curation and analysis. CC, DD, LS, SP, WP, GF, SM, JS, PW, WN-C-N, and IA: methodology. CC: project administration and writing—original draft. CC, DD, SP, and PW: resources. CC, DD, LS, SP, WP, GF, NB, JH, CS, GT, VS, MC, FC, SM, JS, PW, WN-C-N, and IA: visualization. All authors contributed to the article and approved the submitted version.

## Supplementary material

Supplementary Table 1 Dataset releases and recaptures.

Supplementary Table 2 Dataset rehabilitation and release.

Supplementary Table 3 Financial costs.

Supplementary Table 4 Health tests.

Supplementary Table 5 Deaths and pathology.

Supplementary Table 6 Atoxoplasma matrix.

## References

[1] Gray TNE Marx N Khem V Lague D Nijman V Gauntlett S. Holistic management of live animals confiscated from illegal wildlife trade. J Appl Ecol. (2017) 54:726–30. 10.1111/1365-2664.12916

[2] Ribeiro J Reino L Schindler S Strubbe D Vall-Lloseras M Araújo MB. A trends in legal and illegal trade of wild birds: a global assessment based on expert knowledge. Biodivers Conserv. (2019) 28:3343–69. 10.1007/s10531-019-01825-5

[3] Parry-Jones R Allan C. Tackling Wildlife Crime. Annual review 2019–2021. WWF and TRAFFIC, Gland, Switzerland. (2021). Available online at: https://wwfint.awsassets.panda.org/downloads/wwf_tackling_wildlife_crime_review_2019_2021_v4singles__1_.pdf (accessed May 9, 2022).

[4] Rossi A. Uganda Wildlife Trafficking Assessment. Traffic International, Cambridge, United Kingdom. (2018). Available online at: https://www.traffic.org/site/assets/files/8460/uganda_wildlife_assessment.pdf (accessed May 10, 2022).

[5] Destro GFC De Marco P Terribile LC. Comparing environmental and socioeconomic drivers of illegal capture of wild birds in Brazil. Environ Conserv. (2019) 47:1–6. 10.1016/j.pecon.2018.03.003

[6] Krishnasamy K Zavagli M. Southeast Asia: At the heart of wildlife trade. TRAFFIC edition, Southeast Asia Regional Office, Selangor, Malaysia. (2020). Available online at: https://webmail.ufrgs.br/chasque/?_task=mail&_frame=1&_mbox=INBOX&_uid=52600&_part=3&_action=get&_extwin=1 (accessed May 9, 2022).

[7] Alves RRN Lima JRF Araújo HF. The live bird trade in Brazil and its conservation implications: an overview. Bird Conserv Int. (2013) 23:53–65. 10.1017/S095927091200010X

[8] Chng SCL Guciano M Eaton JA. In the market for extinction: Sukahaji, Bandung, Java, Indonesia. BirdingAsia. (2016) 26:22–8. Available online at: https://www.traffic.org/site/assets/files/2393/bandung-birdingasia-article.pdf (accessed April 20, 2022).29922508

[9] Harris J Tingley M Hua F Yong D Adeney J Lee T et al. Measuring the impact of the pet trade on Indonesian birds. Conserv Biol. (2017) 31:394–05. 10.1111/cobi.12729 28146342

[10] Charity S Ferreira JM. Wildlife trafficking in Brazil. Traffic International, Cambridge, United Kingdom. (2020). Available online at: https://www.traffic.org/site/assets/files/13031/brazil_wildlife_trafficking_assessment.pdf (accessed March 15 2022).

[11] Cruz CEF Soares CES Hirt GB Wagner PGC Andretta I Neto WN-C. Wild animals housed in the IBAMA Triage Center in Southern Brazil, 2005–2021: a glimpse into the endless conflicts between man and other animals. Ethnobiol Conserv. (2022) 11:29. 10.15451/ec2022-09-11.28-1-29

[12] ICMBIO. (2018). Livro vermelho da fauna Brasileira ameaçada de extinção. Available online at: https://www.icmbio.gov.br/portal/images/stories/comunicacao/publicacoes/publicacoes-diversas/livro_vermelho_2018_vol1.pdf (accessed April 20, 2022).

[13] IUCN. (2022). The IUCN Red List of Threatened Species. Gland, Switzerland: Available online at: https://www.iucnredlist.org (accessed April 20, 2022).

[14] Mittermeier JC Oliveros CH Haryoko T Irham M Moyle RG. An avifaunal survey of three Javan volcanoes—Gn Salak, Gn Slamet and the Ijen highlands. BirdingAsia. (2014) 22:91–100. Available online at: https://www.researchgate.net/publication/274387607_An_avifaunal_survey_of_three_Javan_volcanoes–Gn_Salak_Gn_Slamet_and_the_Ijen_highlands (accessed May 9, 2022).

[15] Chng SCL Saaban S Weichit A Krishnasamy K. Smuggled for its song—The Trade in Malaysia's Oriental Magpie-robins. TRAFFIC, Southeast Asia Regional Office, Petaling Jaya, Selangor, Malaysia. (2021). Available online at: https://www.traffic.org/site/assets/files/13547/smuggled_for_its_song_oriental_magpie_robin_trade_malaysia_en_report_5_march.pdf (accessed May 9, 2022).

[16] Bowler DE Heldbjerg H Fox AD de Jong M Böhning-Gaese K. Long-term declines of European insectivorous bird populations and potential causes. Conserv Biol. (2019) 33:1120–30. 10.1111/cobi.13307 30912605

[17] Rosenberg KV Dokter AM Blancher PJ Sauer JR Smith PA Stanton JC. Decline of the North American avifauna. Science. (2019) 366:120–4. 10.1126/science.aaw1313 31604313

[18] Hooper DU Adair EC Cardinale BJ Byrnes J Hungate B Matulich K et al. A global synthesis reveals biodiversity loss as a major driver of ecosystem change. Nature. (2012) 486:105–8. 10.1038/nature11118 22678289

[19] Whelan CJ Sekercioglu CH Wenny DJ. Why birds matter: from economic ornithology to ecosystem services. J Ornithol. (2015) 156:227–38. 10.1007/s10336-015-1229-y

[20] Tchoumbou MA Malange EFN Tiku CT Tibab B Fru-Cho J Tchuinkam T. Response of understory bird feeding groups to deforestation gradient in a tropical rainforest of Cameroon. Trop Conserv Sci. (2020) 13:1–12. 10.1177/1940082920906970

[21] IUCN. Guidelines for the Placement of confiscated Animals. Gland, Switzerland. (2000). Available online at: https://portals.iucn.org/library/sites/library/files/documents/2002-004.pdf (accessed April 20, 2022).

[22] IUCN. Guidelines for the Management of Confiscated Live Organisms. Maddison N, editor. Gland, Switzerland. (2019): pp. 38. 10.2305/IUCN.CH.2019.03.en (accessed April 20, 2022).

[23] Kock RA Woodford MH Rossiter PB. Disease risks associated with the translocation of wildlife. Rev Sci Tech - Off Int Epizoot. (2010) 29:329–50. 10.20506/rst.29.2.1980 20919586

[24] Ferreira JM,. (2012). Contribuição da genética de populações à investigação sobre o tráfico de fauna no Brasil: desenvolvimento de microssatélites e análise da estrutura genética em *Paroaria dominicana* e *Saltator similis* (Aves: Passeriformes: Thraupidae). [Doctoral dissertation]. [São Paulo (SP)]: Instituto de Biociências da Universidade de São Paulo. Available online at: https://teses.usp.br/teses/disponiveis/41/41131/tde-17122012-200300/publico/Juliana_Ferreira.pdf

[25] Whitlock R Stewart GB Goodman SJ Piertney SB Butlin RK Pullin AS. A systematic review of phenotypic responses to between-population outbreeding. Environ Evid. (2013) 2:13. 10.1186/2047-2382-2-13

[26] Jakob-Hoff RM MacDiarmid SC Lees C Miller PS Travis D Kock R. Manual of Procedures for Wildlife Disease Risk Analysis. World Organization for Animal Health, Paris, 160 pp. Published in association with the International Union for Conservation of Nature and the Species Survival Commission. (2014). Available online at: file:///C:/Users/Claudio/Downloads/Manual_of_procedures_for_wildlife_diseas.pdf (accessed February 18, 2022).

[27] Cruz CEF Funkler GR Zani ALS Wagner PGC Andretta I Segura LN. preliminary assessment of the potential health and genetic impacts in releasing confiscated passerines into the wild: a reduced-risk approach. Front Vet Sci. (2021) 8:679049. 10.3389/fvets.2021.679049 34708099 PMC8542797

[28] Destro GFC De Marco P Terribile LC. Threats for bird population restorations: a systematic review. Perspect Ecol Conserv. (2018) 16:68–73. 34269510

[29] Macias C Parás A González JJ Enkerlin E Ritchie B Stone E Lamberski N Ciembor D. Release of confiscated amazon parrots in Mexico. PsittaScene. (2003) 15:2–4. Available online at: https://issuu.com/worldparrottrust/docs/15_3_aug_03 (accessed November 3, 2022).

[30] Lankester F Conzo G. A 2nd chance for confiscated greys. PsittaScene. (2008) 20:3–4. Available online at: https://issuu.com/worldparrottrust/docs/ps_20_1_feb_08?mode=mobile (accessed November 3, 2022).

[31] Richardson K Castro IC Brunton DH Armstrong DP. Not so soft? Delayed release reduces long-term survival in a passerine reintroduction. Oryx. (2013) 49:535–41. 10.1017/S0030605313001014

[32] Collar NJ Lierz M Price MRS Wirth RZ. Release of confiscated and captive-bred parrots: is it ever acceptable? Oryx. (2015) 49:202–3. 10.1017/S0030605314001136

[33] Ortiz D Capllonch P. Distribución y migración de *Sporophila c. caerulescens* em Sudamérica. Rev Bras Ornitol. (2007) 15:377–385. Available online at: http://revbrasilornitol.com.br/BJO/article/view/3004/pdf_470 (accessed February 18, 2022).

[34] Cruz CEF Wagner PGC Driemeier D Andretta I. Live decoys: an old but effective tool for attracting, capturing, and studying free-living passerines. Eur J Wildl Res. (2022) 68:24. 10.1007/s10344-022-01571-0

[35] Rinaldi L Coles CG Maurelli MP Musella V Cringoli G. Calibration and diagnostic accuracy of simple flotation, McMaster and FLOTAC for parasite egg counts in sheep. Vet Parasitol. (2011) 177:345–52. 10.1016/j.vetpar.2010.12.010 21216533

[36] Duszynski DW Wilber PG. A Guideline for the preparation of species descriptions in the eimeriidae. J Parasitol. (1997) 83:333–36. 10.2307/3284470 9105325

[37] De Carli GA. Parasitologia Clínica: Seleção De Métodos E Técnicas De Laboratório Para O Diagnóstico Das Parasitoses Humanas. 2nd ed. São Paulo: Atheneu (2007), p. 59–62.

[38] Little SE Kelly LS Norton TM Terrell SP. Developing diagnostic tools to further our understanding of Atoxoplasma species. Proc Assoc Avian Vet. (2001) 3:157–9.

[39] Altschul SF Gish W Miller W Myers EW Lipman DJ. Basic local alignment search tool. J Mol Biol. (1990) 5:215–403. 10.1016/s0022-2836(05)80360-2 2231712

[40] Thompson JD Higgins DG Gibson TJ CLUSTAL W. improving the sensitivity of progressive multiple sequence alignment through sequence weighting, position-specific gap penalties and weight matrix choice. Nucleic Acids Res. (1994) 22:4673–80. 10.1093/nar/22.22.4673 7984417 PMC308517

[41] Cruz CEF Cerva C Andretta I. Financial costs of conserving captive-bred wild birds. Zool Gart. (2016) 85:354–62. 10.1016/j.zoolgart.2016.08.003

[42] Cruz CEF Fredo G Casagrande R Oliveira L Rolim V Marques S. Eucoleus contortus parasitism in captive-bred Valley quail *Callipepla californica* (Shaw, 1798): disease and control. Zool Gart. (2016) 85:152–9. 10.1016/j.zoolgart.2016.01.008

[43] Cruz CEF Marques SMT Andretta I. Endoparasites observed within invertebrates used as live food items for captive wild birds: overview and potential risks. ZooBiol. (2019) 38:384–8. 10.1002/zoo.21500 31206830

[44] Grogan A Kelly A. A review of the RSPCA research in wildlife rehabilitation. Vet Rec. (2013) 172:211–4. 10.1136/vr.101139 23436601

[45] Molina-López RA Mañosa S Torres-Riera A Pomarol M Darwich L. Morbidity, outcomes and cost-benefit analysis of wildlife rehabilitation in Catalonia (Spain). PLoS ONE. (2017) 12:e0181331. 10.1371/journal.pone.0181331 28719647 PMC5515437

[46] Bušina T Pasaribu N Hlavsa T Czerneková V Kouba M. An experimental release of rehabilitated wild-caught Sumatran Laughingthrush *Garrulax bicolor*: assessment of post-release survival and dispersal *via* radio-telemetry, North Sumatra, Indonesia. Ornithol Sci. (2018) 17:135–47. 10.2326/osj.17.135

[47] D'Angelo VS Grajal A. Successful reintroduction of captive-raised yellow-shouldered amazon parrots on Margarita Island, Venezuela. Conserv Biol. (2008) 12:430–41. 10.1111/j.1523-1739.1998.96261.x

[48] Cristinacce A Handschum M Switzer RA Cole RE Tatayah V Jones CG Bell D. The release and establishment of Mauritius fodies *Foudia rubra* on Ile aux Aigrettes, Mauritius. Conserv Evid. (2009) 6:1–15. Available online at: https://www.researchgate.net/publication/237272437_The_release_and_establishment_of_Mauritius_fodies_Foudia_rubra_on_Ile_aux_Aigrettes_Mauritius (accessed August 12, 2022).

[49] Walters JR Derrickson SR Fry DM Haig SM Marzluff JM Wunderle JM Jr. Status of the California Condor (*Gymnogyps californianus*) and efforts to achieve its recovery. The Auk. (2010) 127:969–1001. 10.1525/auk.2010.127.4.969

[50] White TH Jr Collar NJ Moorhouse RJ Sanz V Stolen ED Brightsmith DJ. Psittacine reintroductions: common denominators of success. Biol Conserv. (2012) 148:106–15. 10.1016/j.biocon.2012.01.044

[51] Soorae PS. Global conservation translocation perspectives: 2021. 7^th^ ed. Case studies from around the globe. Gland, Switzerland: IUCN SSC Conservation Translocation Specialist Group, Environment Agency - Abu Dhabi and Calgary Zoo, Canada. xiv + 353 p. Available online at: https://portals.iucn.org/library/sites/library/files/documents/2021-007-En.pdf (accessed October 15, 2022).

[52] Lee JGH Chng SCL Eaton JA. (2016) Conservation strategy for Southeast Asian songbirds in trade. Recommendations from the first Asian Songbird Trade Crisis Summit 2015 held in Jurong Bird Park, Singapore, 27–29 September (2015). Available online at: https://www.traffic.org/site/assets/files/2275/conservation-strategy-for-southeast-asian-songbirds-in-trade.pdf (accessed June 11, 2022).

[53] Winfree R Fox JW Williams NM Reilly JR Cariveau DP. Abundance of common species, not species richness, drives delivery of a real-world ecosystem service. Ecol Lett. (2015) 18:626–35. 10.1111/ele.12424 25959973

[54] Baker DJ Garnett ST O'Connor J Ehmke G Clarke RH Woinarski JCZ. Conserving the abundance of non-threatened species. Conserv Biol. (2019) 33:319–28. 10.1111/cobi.13197 30047186

[55] Gardner CJ Bicknell JE Baldwin-Cantelho W Struebing MJ Davies ZG. Quantifying the impacts of defaunation on natural forest regeneration in a global meta-analysis. Nat Commun. (2019) 10:4590. 10.1038/s41467-019-12539-1 31611554 PMC6791894

[56] Karsten P. Pekin Robins and Small Softbills Management and Breeding. Surrey, Canada: Hancock House Publishers (2007). 251 p.

[57] Cruz CEF Oliveira L Boabaid F Zimermann F Stein G Marks F. Management, breeding, and health records from a captive colony of Pekin robins (*Leiothrix lutea*), 2001–2010. J Zoo Wildl Med. (2011) 42:451–9. 10.1638/2011-0050.1 22950318

[58] Bostwick K. Feathers and plumages. In:Lovette IJ Fitzpatrck JW, editors. The Cornell Lab of Ornithology. Handbook of Bird Biology, 3rd ed. Chichester: Wiley (2016). p. 101–147.

[59] Tobalske BW. Avian flight. In:Lovette IJ Fitzpatrck JW, editors. The Cornell Lab of Ornithology. Handbook of Bird Biology, 3rd ed. Chichester: Wiley (2016). p. 149–167.

[60] Hoff FH Jr. Conditioning, Spawning, and Rearing of Fish With Emphasis on Marine Clownfish. (1996). Florida: Aquaculture Consultants Inc. 212 p.

[61] Echenique JVZ Soares MP Albano ANP Bandarra PM Schild AL. Diseases of wild birds in southern Rio Grande do Sul, Brazil. Pesq Vet Bras. (2020) 40:121–8. 10.1590/1678-5150-PVB-6409

[62] Oliveira LGS Lipinski GP Lorenzett MP Rolim VM Marques SMT Driemeier D. Causes of bird losses recorded in a captive-bred wild bird flock between 2011 and 2015. Cienc Rural. (2017) 47:e20160903. 10.1590/0103-8478cr20160903

[63] Bub H. Bird Trapping and Bird Banding: A Handbook for Trapping Methods All over the World. New York: Cornell Univ Press. (1991). 330 p.

[64] Malley D. Handling and clinical examination of psittacine birds. In Pract. (1996) 18:302–11. 10.1136/inpract.18.7.302

[65] Alexander DJ Senne DA. Newcastle Disease, Other Avian Paramyxoviruses, and Pneumovirus Infections. In: Saif YM, Fadly AM, Glisson JR, McDougald LR, Nolan LK, Swayne DE, editors. Diseases of Poultry. Iowa State University Press (2008). p. 75–100.

[66] Thomas NJ Hunter DB Atkinson CT. Infectious Diseases of Wild Birds. 1st ed. Iowa: Blackwell Publishing (2007). 491 p.

[67] Oliveira-Júnior JG Portz C Loureiro BO Schiavo PA Fedullo LPL Mazur C. Vírus da doença de Newcastle em aves não vacinadas no estado do Rio de Janeiro. Cienc Rural. (2003) 33:381–3. 10.1590/S0103-84782003000200033

[68] Camenisch G Bandli R Hoop R. Monitoring of wild birds for Newcastle disease virus in Switzerland using real time RT-PCR. J Wildl Dis. (2008) 44:772–6. 10.7589/0090-3558-44.3.772 18689670

[69] Cunha MPV Guimarães MB Davies YM Milanelo L Knöbl T. Bactérias gram-negativas em cardeais (*Paroaria coronata* e *Paroaria dominicana*) apreendidos no tráfico de animais silvestres. Braz J Vet Res Anim Sci. (2016) 53:107–11. 10.11606/issn.1678-4456.v53i1p107-111

[70] Dhondt AA DeCoste J Ley DH Hochachka WM. Diverse wild bird host range of *Mycoplasma gallisepticum* in Eastern North America. PLoS ONE. (2014) 3:e103553. 10.1371/journal.pone.0103553 25061684 PMC4111589

[71] Ewen JG Acevedo-Whitehouse K Alley MR Carraro C Sainsbury AW Swinnerton K Woodroffe R. Empirical consideration of parasites and health in reintroduction. In:Ewen JG Armstrong DP Parker KA Seddon PJ, editors. Reintroduction Biology: Integrating Science and Management. 1rst ed. Oxford, UK: Blackwell Publishing (2012). p. 290–335.

[72] Atkinson CT Thomas NJ Hunter DB. Parasitic Diseases of Wild Birds. 1st ed. Iowa, USA: Blackwell Publishing (2008). 595 p.

[73] Cushing TL Schat KA States SL Grodio JL O'Connell PH Buckles EL. Characterization of the host response in systemic isosporosis (Atoxoplasmosis) in a colony of captive American goldfinches (*Spinus tristis*) and house sparrows (*Passer domesticus*). Vet Pathol. (2011) 48:985–92. 10.1177/0300985810391114 21311069

[74] Godoy TNA Matushima ER. A survey of diseases in passeriform birds obtained from illegal wildlife trade in São Paulo City, Brazil. J Avian Med Surg. (2010) 24:199–209. 10.1647/2009-029.1 21046940

[75] Oliveira AR Souza TD Mola JPS Flecherb MC Hiurab E Santos RS. Pathological and molecular characterization of systemic Isosporosis (Atoxoplasmosis) in captive green-winged saltator (*Saltator similis*). Vet Parasithol. (2018) 255:98–101. 10.1016/j.vetpar.2018.04.007 29773145

[76] McOrist S Barton NJ Black DG. Acuaria Skrjabini infection of the gizzard of finches. Avian Dis. (1982) 26:957–90. 10.2307/1589887 7159331

[77] Brown MJ Linton E Rees EC. Causes of mortality among wild swans in Britain. Waterfowl. (1992) 43:70–9.26629647

[78] Oonincx D Dierenfeld E. An investigation into the chemical composition of alternative invertebrate prey. Zoo Biol. (2011) 29:1–15. 10.1002/zoo.20382 21442652

[79] Henao-Duque AM Buitrago GD Peña-Stadlin J Carvallo-Chaigneau F. Outbreaks of *Sarcocystis spp*. in exotic birds kept in captivity at the Cali Zoo (Colombia): case series. Rev MVZ Córdoba. (2022) 27:e2146. 10.21897/rmvz.2146

[80] Shurkin J. News feature: animals that self-medicate. Proc Natl Acad Sci USA. (2014) 111:17339–41. 10.1073/pnas.1419966111 25492915 PMC4267359

[81] Sapolsky RM Romero LM Munck AU. How do glucocorticoids influence stress responses? Integrating permissive, suppressive, stimulatory, and preparative actions. Endocr Rev. (2000) 21:55–89. 10.120/er.21.1.55 10696570

[82] Dickens MJ Delehanty DJ Romero LM. Stress: an inevitable component of translocation. Biol Conserv. (2010) 143:1329–41. 10.1016/j.biocon.2010.02.032

[83] Angelier F Parenteau C Trouvé C Angelier N. Does the stress response predict the ability of wild birds to adjust to short-term captivity? A study of the rock pigeon (*Columbia livia*). R Soc open sci. (2016) 3:160840. 10.1098/rsos.160840 28083117 PMC5210699

[84] Friend M Toweill DE Brownell RL Nettles VF Davis DS Foreyt DJ. Guidelines for proper care and use of wildlife in field research. In:Friend M Franson JC, editors. Field Manual of Wildlife Diseases: General Field Procedures and Diseases of Birds. Maddison, USA: USGS. (1999). p. 53–72. Available online at: https://pubs.er.usgs.gov/publication/2001108

[85] Gresse R Chaucheyras-Durand F Fleury MA Wiele TV Forano E Blanquet-Diot S. Gut microbiota dysbiosis in post-weaning piglets: understanding the keys to health. Trends Microbiol. (2017) 25:851–73. 10.1016/j.tim.2017.05.004 28602521

[86] Videvall E Song SJ Bensch HM Strandh M Engelbrecht A Serfontein N. Early-life gut dysbiosis linked to juvenile mortality in ostriches. Microbiome. (2020) 8:147. 10.1186/s40168-020-00925-7 33046114 PMC7552511

[87] Brunthaler R Teufelbauer N Seaman B Nedorost N Bittermann K Matt J. Trichomonosis in Austrian songbirds—Geographic distribution, pathological lesions and genetic characterization over 9 years. Animals. (2022) 12:1306. 10.3390/ani12101306 35625152 PMC9137778

[88] Latimer KS Rakich PM. Necropsy examination. In:Ritchie BW Harrison GJ Harrison LR, editors. Avian Medicine Principles and Applications. Florida: Wingers Publishing. (1994). p. 355-379.

[89] Candia-Gallardo C Awade M Boscolo D Bugoni L. Rastreamento de aves através de telemetria por rádio e satélite. In:Matter SV Straube FC Accordi IA Piacentini VQ Cândido-JR JF, editors. Ornitologia e Conservação – Ciência Aplicada, Técnicas de Pesquisa e Levantamento. Technical Books Editora, Rio de Janeiro. (2010). p. 257–277.

[90] Parlato E Armstrong DP. Predicting post-release establishment using data from multiple reintroductions. Biol Conserv. (2013) 160:97–104. 10.1016/j.biocon.2013.01.013 22098341

[91] Magroski LM Pessoa AN Lucena WG Loures-Ribeiro A Araújo CB. Where to release birds seized from illegal traffic? The value of vocal analyses and ecological niche modeling. Perspect Ecol Conserv. (2017) 15:91–101. 10.1016/j.pecon.2017.05.001

[92] Tautin J. One hundred years of bird banding in North America. USDA Forest Service Gen. Tech Rep. (2005). PSW-GTR-191:815–816. Available online at: https://www.fs.fed.us/psw/publications/documents/psw_gtr191/psw_gtr191_0815-0816_tatuin.pdf (accessed October 15, 2022).

[93] Thorup K Korner-Nievergelt F Cohen EB Baillie SR. Large-scale spatial analysis of ringing and re-encounter data to infer movement patterns: a review including methodological perspectives. Methods Ecol Evol. (2014) 5:1337–50. 10.1111/2041-210X.12258

[94] EURING. Euring databank contents – numbers of different categories of records for each species. European Union for Bird Ringing. (2020). Available online at: https://euring.org/files/documents/EDB_contents_species_by_record_type_20200115.pdf (accessed November 10, 2022).

[95] Joys AC Clark JA Clark NA Robinson RA. An investigation of the effectiveness of rehabilitation of birds as shown by recoveries. BTO Research Report N° 324. (2003). Available online at: https://www.bto.org/sites/default/files/shared_documents/publications/research-reports/2003/rr324.pdf (accessed October 15, 2022).

[96] Ewen JG Armstrong DP Empson R Makan SJT McInnes K Parker KA. Parasite management in translocations: lessons from a threatened New Zealand bird. Oryx. (2012) 46:446–56. 10.1017/S0030605311001281

[97] Seddon PJ Strauss WM Innes J. Animal translocations: What are they and why do we do them? In:Ewen JG Armstrong DP Parker KA Seddon PJ, editors. Reintroduction Biology: Integrating Science and Management. 1rst ed. Oxford, UK: Blackwell Publishing (2012). p. 1–32.

[98] Fountain K Jeffs C Croft S Gregson J Lister J Evans A. The influence of risk factors associated with captive rearing on post-release survival in translocated Cirl buntings Emberiza cirlus in the UK. Oryx. (2017) 51:332–8. 10.1017/S0030605315001313

[99] Gama TP Sassi R. Aspectos do comércio ilegal de pássaros silvestres na cidade de João Pessoa, Paraíba, Brasil. Gaia Scientia. (2008) 2:1–20. Available online at: https://www.researchgate.net/publication/31515169_Aspectos_do_comercio_Ilegal_de_Passaros_Silvestres_na_Cidade_de_Joao_Pessoa_Paraiba_Brasil (accessed May 14, 2022).

[100] EBird. An online database of bird distribution and abundance [web application]. eBird, Cornell Lab of Ornithology, Ithaca, New York. (2022). Available online at: http://www.ebird.org (accessed May 29 2022).

[101] Mason JR Reidinger RF. Effects of social facilitation and observational learning on feeding behavior of the Red-winged Blackbird (*Agelaius phoeniceus*). The Auk. (1981) 98:778–84.

[102] Segura LN Perelló M Gress NH Ontiveros R. The lack of males due to illegal trapping is causing polygyny in the globally endangered Yellow Cardinal *Gubernatrix cristata*. Rev Bras Ornitol. (2019) 27:40–43. 10.1007/BF03544445

[103] Lindenmayer DB Hoobs RJ. Fauna conservation in Australian plantation forests—A review. Biol Conserv. (2004) 119:151–68. 10.1016/j.biocon.2003.10.028

[104] Brockerhoff EG Jactel H Parrotta JA Quine CP Sayer J. Plantation forests and biodiversity: oxymoron or opportunity? Biodivers Conserv. (2008) 17:925–51. 10.1007/s10531-008-9380-x

[105] Bremer LL Farley KA. Does plantation forestry restore biodiversity or create green deserts? A synthesis of the effects of land-use transitions on plant species richness. Biodivers Conserv. (2010) 19:3893–915. 10.1007/s10531-010-9936-4

[106] Williams DR Clark M Buchanan GM Ficetola GF Rondinini C Tilman D. Proactive conservation to prevent habitat losses to agricultural expansion. Nat Sustain. (2020) 4:314–22. 10.1038/s41893-020-00656-5

[107] Massey JG Hampton S Ziccardi MH. A cost/benefit analysis of oiled wildlife response. Int Oil Soil Conf Proceed. (2005) 1:463–6. 10.7901/2169-3358-2005-1-463

[108] Machado RB Silveira LF da Silva MISG Ubaid FK Medolago CA Francisco MR. Reintroduction of songbirds from captivity: the case of the Great-billed Seed-finch (*Sporophila maximiliani*) in Brazil. Biodivers Conserv. (2020) 29:1613–36. 10.1007/s10531-019-01830-8

